# Energy expenditure estimation from respiration variables

**DOI:** 10.1038/s41598-017-16135-5

**Published:** 2017-11-22

**Authors:** Rahel Gilgen-Ammann, Marcel Koller, Céline Huber, Riikka Ahola, Topi Korhonen, Thomas Wyss

**Affiliations:** 1 Swiss Federal Institute of Sport Magglingen SFISM, Magglingen, Switzerland; 2 Polar Electro, Kempele, Finland

## Abstract

The aim of this study was to develop and cross-validate two models to estimate total energy expenditure (TEE) based on respiration variables in healthy subjects during daily physical activities. Ninety-nine male and female subjects systematically varying in age (18–60 years) and body mass index (BMI; 17–36 kg*m^−2^) completed eleven aerobic activities with a portable spirometer as the criterion measure. Two models were developed using linear regression analyses with the data from 67 randomly selected subjects (50.0% female, 39.9 ± 11.8 years, 25.1 ± 5.2 kg*m^−2^). The models were cross-validated with the other 32 subjects (49% female, 40.4 ± 10.7 years, 24.7 ± 4.6 kg*m^−2^) by applying equivalence testing and Bland-and-Altman analyses. Model 1, estimating TEE based solely on respiratory volume, respiratory rate, and age, was significantly equivalent to the measured TEE with a systematic bias of 0.06 kJ*min^−1^ (0.22%) and limits of agreement of ±6.83 kJ*min^−1^. Model 1 was as accurate in estimating TEE as Model 2, which incorporated further information on activity categories, heart rate, sex, and BMI. The results demonstrated that respiration variables and age can be used to accurately determine daily TEE for different types of aerobic activities in healthy adults across a broad range of ages and body sizes.

## Introduction

Physical activity helps to prevent chronic diseases and premature death, for example by augmenting energy metabolism^1^. In the prevention or treatment of several lifestyle-related diseases, the assessment of daily energy expenditure plays an important role in regulating body weight^2^. Total energy expenditure (TEE) in humans consists of the basal metabolic rate, the thermic effect of food, and the energy expenditure caused by physical activity^3^.

Despite the importance of the appropriate amount of daily TEE, accurate assessment of TEE in free-living conditions remains difficult^4–6^. For instance, self-reported questionnaires or seven-day physical activity recalls intended to evaluate TEE were shown to either over- or underestimate TEE by up to 60%^7,8^. There is a clear need for measurement tools that allow for the objective monitoring of individuals’ TEE. A range of accelerometer- or heart-rate-based activity monitors are on the market that claim to obtain TEE or activity energy expenditure^9^. Their outputs have been shown to produce relatively small to moderate mean differences between the estimated and measured energy expenditures^10–13^. Yet, in order to minimize individual errors, these methods may require additional information like activity task recognition, subjects’ anthropometrics, calibration, or users’ training statuses^5,6^.

Another approach is the measurement of respiration variables^14,15^. Currently, there is a fair amount of newer developments that aim towards the assessment of respiration variables in free-living individuals^16^. These wearables include sensors such as a respiration electrode patch that operates via impedance plethysmography or bioimpedance and are incorporated into for example, smart t-shirts^17–19^. Already in the middle of the 20^th^ century, a linear relationship between pulmonary ventilation and TEE was demonstrated^20^. Several previous studies highlighted that TEE can be estimated based on pulmonary ventilation only^15,20–22^ or in addition to heart rate^23,24^ and/or body weight^25^. However, the prediction models based on respiration variables have typically been evaluated using the same individuals from whom the original equations were derived^15,23,24^, and the data obtained under laboratory conditions were primarily from sitting and gait activities^15,24,25^. In addition, the previous research was generally based on small sample sizes that were restricted to specific subgroups, such as male participants or active people^13,15,22,24^. Consequently, little is known about the precision offered by TEE predictions based on respiration variables when used under free-living conditions or during different intensities. It is also unknown whether the TEE estimations are valid for a broad population (such as in younger to older people, male and female adults, or under-, normal-, and overweight people). Such evidence would be necessary in order to justify more effort into the development of portable devices measuring respiration variables for activity monitoring. Therefore, the aim of this study was the calculation and cross-validation of two models estimating daily TEE from respiration variables, heart rate, and anthropometrics for different types of aerobic activities in a broad population group.

## Materials and Methods

### Subjects

Healthy male and female volunteers were recruited to participate in this study. Anthropometrics including age, sex, height, and weight were obtained by self-report to ensure that the sample would consist of a broad range of ages and body sizes and that the final models would therefore be applicable to a broader population. Exclusion criteria were an age above 60 years of age or body mass index (BMI) > 36 kg*m^−2^. Potentially eligible participants were screened using the physical activity readiness questionnaire (PAR-Q) to assess whether the subjects could do all the exercises without risk^26^. Participants who answered yes to any PAR-Q question or took any medication affecting the heart or metabolism were excluded from the study. In total, 113 subjects were recruited to participate in this study. All participants signed an informed consent form prior to data collection. The final study sample consisted of 99 participants (Table 1). The data of 14 subjects were excluded due to technical problems with the reference device (2.6%) or the heart rate monitor (9.7%).

**Table 1:** Characteristics of the final study sample obtained in the laboratory presented as mean ± standard deviation.

	Total N = 99	Men N = 50	Female N = 49
Age [years]	40.2 ± 11.1	40.7 ± 11.3	39.8 ± 10.9
Height [m]	1.74 ± 0.09	1.80 ± 0.07	1.68 ± 0.06
Weight [kg]	75.6 ± 17.2	83.4 ± 17.1	67.6 ± 13.2
Body mass index [kg*m^−2^]	24.8 ± 4.8	25.7 ± 4.5	24.0 ± 4.9
VO_2_max [ml*min*kg]	45.5 ± 10.0	48.7 ± 9.2	42.1 ± 9.6

The study and consent form were reviewed and approved by the ethics commission of the Canton Berne. All experiments were performed in accordance with relevant guidelines and regulations.

### Experimental protocol

On two test days separated by one week, the participants had not consumed caffeine or participated in exercise for the previous 12 hours. On day 1, maximal oxygen uptake (VO_2_max) was measured by an incremental test in running to volitional exhaustion applying the adapted Bruce protocol ramp test^27,28^. On day 2, data collection was completed with each participant individually, and each performed eleven aerobic activities that were categorised as sitting, household, cyclic, and anti-cyclic (Table 2). The latter comprised strength training (biceps curls with individual weights, sit-ups, lunges, and push-ups), tennis play with a partner, and a soccer course (including drippling, sprinting with/without the ball, passing the ball, and shooting). The configuration of the tasks was designed to be as realistic as possible. Each activity lasted four minutes with a one-minute resting time after the transition from the previous to the next activity. The order of the activities was predetermined, starting with the anticipated lowest task intensity (Table 2). Task intensities were self-selected to represent individual habits^13^. Walking and running speeds averaged 4.3 km*h^−1^ (ranging from 3.0–5.0 km*h^−1^) and 9.8 km*h^−1^ (ranging from 7.5–12.0 km*h^−1^), respectively.

**Table 2:** The eleven activity tasks categorised and presented in the order of execution and its intensity as mean ± standard deviation.

Activity task	% VO_2_max
*Sitting*	
Office work	10.7 ± 3.2
Stroop test	10.0 ± 2.3
*Household duties*	
Cleaning table	22.9 ± 5.4
Floor sweeping	28.4 ± 6.9
Tidying up	32.0 ± 7.5
*Cyclic activities*	
Cycling on a cycle ergometer	49.0 ± 7.1
Walking flat on a treadmill	31.9 ± 7.1
Running flat on a treadmill	74.3 ± 9.2
*Anti-cyclic sport activities*	
Strength training*	41.8 ± 6.0
Tennis play	69.0 ± 14.0
Soccer course**	82.8 ± 13.0

*Note*. *self-guided biceps curls, sit-ups, lunges, and push-ups; **including drippling, sprinting with/without the ball, passing the ball, and shooting.

### Instruments

A portable open-circuit metabolic system (MetaMax 3B; Cortex Biophysik, Leipzig, Germany) was used to obtain measures of oxygen consumption, carbon dioxide production, respiratory volume, and respiratory frequency^29,30^. The equipment was calibrated prior to each measurement according to the manufacturer’s instructions including ambient air pressure, gas, and volume. The device was mounted on the participant with a face mask and a chest harness. Heart rate assessment was accomplished by means of a chest strap (WearLink wind, Polar Electro Oy, Kempele, Finland). Running and cycling were performed on a treadmill (Mercury; h/p/cosmos sports & medical GmbH, Nussdorf-Traunstein, Germany) and a cycle ergometer (Ergoselect 200; Ergoline GmbH, Bitz, Germany), respectively.

### Data analysis

Two models were developed for the estimation of TEE: the “simpler” Model 1, without the incorporation of known activity tasks, and Model 2, which included these tasks. It has previously been shown that activity recognition increases the accuracy of TEE estimation; however, one disadvantage is that it requires valid measurement systems to obtain activity tasks^5^.

For the development and cross-validation of the models, the sample was randomly assigned to two groups. To develop robust prediction equations, the sample was balanced with respect to sex, four age categories (18–29, 30–39, 40–49, and 50–60 years of age), and four BMI categories (17–19.9, 20–24.9, 25–29.9, and 30–36 kg*m^−2^). Thereafter, two-thirds of each “sex-age-BMI” category were randomly allocated to the developmental group (N = 67, 67.7%), while the remaining participants served as the cross-validation group (N = 32, 32.3%). This design may be a reasonable balance between optimizing bias and variability^31^, and therefore, was also applied in related research^32^.

Breath-by-breath data for oxygen uptake, carbon dioxide emission, respiratory volume, and respiratory frequency were collected; from the four-minute activities, the average values for one minute measured from 2:45 to 3:45 were used to calculate TEE [kJ*min^−1^] using Péronnet’s formula^33^. This approach is commonly accepted for estimating TEE during aerobic or submaximal intensities^34,35^. However, the formula does not hold for anaerobic activities, as TEE was shown to be significantly underestimated^36,37^. Therefore, the focus in the present study was on aerobic tasks. To ensure the limitation to aerobic data in the developmental and validation groups, the measurements with respiratory quotients > 1 were excluded in both groups^38^. From cyclic and anti-cyclic activities we removed a total of 105 of 737 (14%) data points for the developmental group and 42 of 352 (12%) for the validation group.

### Statistical analysis

Statistical analyses were performed using Excel 2011 (Microsoft, Redmond, WA) and SPSS 22.0 (SPSS, Inc. Chicago, IL), and the results were considered to be significant if *p* ≤ 0.05. Using the data from the developmental group, two models were determined to reflect the best set of predictors. To investigate Model 1, a backward multiple linear regression was performed with TEE as the dependent variable and respiratory volume, respiratory frequency, sex, BMI, age, and heart rate as independent variables. To compute Model 2, a separate backward multiple linear regression equation was applied for each of the four activity categories with the aforementioned independent variables, prior to summarization in one regression equation. In the case of multicollinearity with respiratory volume or respiratory frequency (target variables) or non-significant prediction of TEE within the models, the relevant variable was excluded from that particular regression analysis.

Thereafter, the two resulting regression equations were applied as Model 1 and Model 2 to the data from the cross-validation group in order to evaluate their accuracy in the estimation of TEE. Equivalence testing was performed to determine whether the estimations were significantly equivalent to the criterion measure^10,39,40^. The estimates were considered to be equivalent if the 95% confidence interval for the absolute mean error of the estimated TEE fell into the proposed equivalence zone (±5%) of the measured TEE^39,40^. Bland-and-Altman plots with corresponding 95% limits of agreement were used to calculate and visualize systematic differences in TEE predictions^41^. Lastly, the root mean square errors and the Pearson correlation coefficients (*r*) were calculated.

## Results

The developmental and validation groups did not differ in terms of age (40.4 ± 10.7 years and 39.9 ± 11.8 years, respectively, *p* = 0.536), BMI (24.7 ± 4.6 kg*m^−2^ and 25.1 ± 5.2 kg*m^−2^, respectively, *p* = 0.259), sex (50.0% and 49.2% female, respectively, *p* = 0.816), and training status (VO_2_max: 45.0 ± 11.5 and 45.7 ± 9.1 ml*kg*min^−1^, respectively, *p* = 0.349).

### Linear regression analyses

For the calculation of Model 1, the variable heart rate had to be excluded due to its multicollinearity with respiratory volume (*r* = 0.812, *p* < 0.001). The sex and BMI variables were also excluded from Model 1 due to non-significant prediction (Equation 1; Table 3). To determine Model 2, the following variables were excluded due to non-significant prediction: the variable age for the sitting and household activities, BMI for the cyclic activities, and respiratory frequency, heart rate, and BMI for the anti-cyclic activities (Equation 2; Table 3).

1 $${\rm{TEE}}[{\mathrm{kJ}\ast \min }^{-1}]=7.473+0.822\ast {\rm{RV}}-0.265\ast {\rm{RF}}-0.055\ast {\rm{age}}$$

2 $$\begin{array}{c}{\rm{TEE}}[{\mathrm{kJ}\ast \min }^{-1}]=\\ \,\,\,\,\,\,\,\,\,if\,sitting\,activity\\ -1.401+0.656\ast {\rm{RV}}-{\rm{0.079}}\ast {\rm{RF}}+0.021\ast {\rm{HR}}-0.351\ast \mathrm{sex}+0{\rm{.55}}\ast {\rm{BMI}}\\ \,\,\,\,\,\,\,\,\,\,if\,household\,activity\\ -2.681+0.825\ast {\rm{RV}}-{\rm{0.117}}\ast {\rm{RF}}+0.032\ast {\rm{HR}}-0.784\ast \mathrm{sex}+0{\rm{.53}}\ast {\rm{BMI}}\\ \,\,\,\,\,\,\,\,\,\,if\,cyclic\,activity\\ 6.714+0.828\ast {\rm{RV}}-0.330\ast {\rm{RF}}+0.39\ast {\rm{HR}}-0.067\ast {\rm{age}}\\ \,\,\,\,\,\,\,\,if\,anti-cyclic\,activity\\ 9.302+0.667\ast {\rm{RV}}-2.180\ast {\rm{sex}}-0.078\ast {\rm{age}}\end{array}$$

when RV is respiratory volume, RF is respiratory frequency, HR is heart rate, BMI is body mass index, and sex is indicated by 0 for male and 1 for female.

**Table 3:** Characteristics of the regression models.

	*R*	*R* ^2^	Adjusted *R* ^2^	SEE
Model 1	0.985	0.970	0.970	3.421
Model 2				
*Sitting*	0.926	0.857	0.851	0.790
*Household*	0.969	0.938	0.937	1.500
*Cyclic*	0.985	0.970	0.969	3.089
*Anti-cyclic*	0.964	0.929	0.927	4.862

*Note*. SEE = Standard error of the estimate.

### Validation

**Figure 1:**
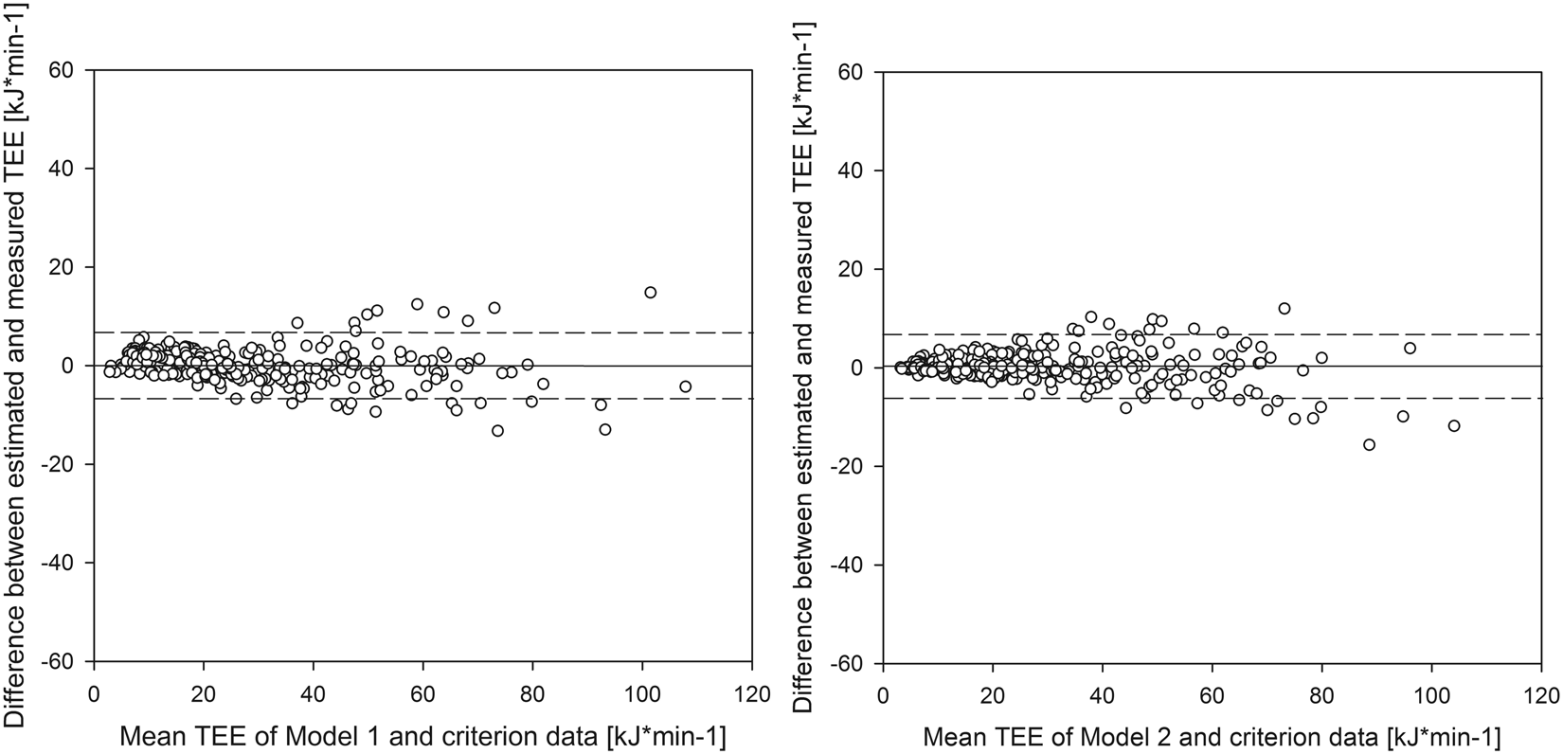
Bland-and-Altman plots of the total energy expenditure (TEE) obtained during different activities: Model 1 vs. criterion measure (left) and Model 2 vs. criterion measure (right). The solid lines represent the systematic bias; the dashed lines represent the limits of agreement (systematic bias ± 1.96*SD).

The calculated mean TEE from the criterion measure, from Model 1 and from Model 2 for each activity task is presented in Table 4. The mean TEE of the criterion measure was 28.35 kJ*min^−1^, of which 5% ( ± 1.42 kJ*min^−1^) was used to determine the interval of tolerable difference. Model 1 resulted in a mean estimated TEE of 28.41 kJ*min^−1^ and an absolute difference from the reference of 0.06 kJ*min^−1^ with limits of agreement of ± 6.83 kJ*min^−1^ (Table 5; Fig. 1). Equivalence testing showed that the criterion data and the values estimated by the regression in Model 1 were significantly equivalent. Since the reported 95% confidence interval (−0.33, + 0.45) for the difference between the estimated TEE from the regression Model 1 and the criterion TEE were completely within the interval of tolerable difference (−1.42, + 1.42), the estimated and the measured TEE can be declared equivalent at the 0.025 significance level.

**Table 4:** The calculated total energy expenditure in kJ*min^−1^ for each activity task as mean ± standard from the criterion measure, the Model 1 and the Model 2.

Activity task	Criterion measure	Model 1	Model 2
*Sitting*			
Office work	7.4 ± 2.1	9.3 ± 2.8*	7.6 ± 2.2^¥^
Stroop test	7.3 ± 1.9	8.7 ± 2.7*	7.6 ± 2.0^¥^
*Household duties*			
Cleaning table	16.5 ± 4.7	17.6 ± 4.9*	17.5 ± 5.2*
Floor sweeping	19.3 ± 5.6	19.2 ± 5.2	19.4 ± 5.5
Tidying up	21.2 ± 5.0	20.9 ± 4.6	21.3 ± 5.0
*Cyclic activities*			
Cycling on a cycle ergometer	37.0 ± 6.6	35.7 ± 6.3	37.3 ± 6.4^¥^
Walking flat on a treadmill	21.1 ± 4.0	20.6 ± 3.7	21.9 ± 4.2*^¥^
Running flat on a treadmill	57.3 ± 14.6	54.8 ± 13.7	57.3 ± 13.7^¥^
*Anti-cyclic sport activities*			
Strength training	32.2 ± 8.7	32.2 ± 9.5	32.8 ± 8.6
Tennis play	49.1 ± 13.7	50.3 ± 13.6	50.0 ± 11.9
Soccer course	65.2 ± 20.2	64.2 ± 20.7	62.2 ± 18.0
**TOTAL**	**28.4 ± 20.3**	**28.4 ± 19.6**	**28.6 ± 19.7**

*Note*. *significant (*p* < 0.05) difference to criterion; ^¥^significant (*p* < 0.05) difference between Model 1 and 2.

**Table 5:** Concurrent validity of the two regression models with the criterion measure.

	Model 1	Model 2
Mean TEE [kJ*min^−1^]	28.411	28.553
Absolute difference to reference [kJ*min^−1^]	0.062	0.204
Relative difference to reference (%)	0.22	0.72
RMSE [kJ*min^−1^]	3.480	3.241
*r* (*p-*value)	0.985 (<0.001)	0.987 (<0.001)

TEE = total energy expenditure; RMSE = root mean square error; *r* = Pearson correlation coefficient.

Model 2 estimated TEE with a bias of 0.20 kJ*min^−1^ with ± 6.35 kJ*min^−1^ limits of agreement (Table 5; Fig. 1). The TEE values calculated by Model 2 were also significantly equivalent to the criterion data. The 95% confidence interval (−0.16, + 0.57) for the difference between the estimated TEE from Model 2 and the criterion TEE was within the equivalence zone (−1.42, +1.42).

## Discussion

This study presents two models based on respiration variables, heart rate, and anthropometrics to estimate aerobic TEE in a broad population under free-living conditions. The accuracy of the two models was evaluated by comparing the estimated TEE with that of a portable spirometer. The findings suggest very high concordance between the methods on the basis of statistical analyses. With relative deviations from the criterion measure of 0.2 ± 12.3% and 0.7 ± 11.4% in Model 1 and Model 2, respectively, the models were significantly equivalent to the criterion. The accuracy of our models was similar to or higher than that of previous studies investigating TEE estimations. For instance, cross-sectional time series models based on heart rate, physical activity measured by accelerometry, and time-invariant covariates predicted TEE with a mean error of 0.9 ± 10.3%^42^. Other models were shown to be less accurate; for example, Rothney *et al*.^43^ validated an arterial neural network model based on acceleration data obtained at the hip and stated a mean difference of 4.5 ± 3.6% compared to the measured TEE. Similarly, an error in TEE prediction of 5% based on pulmonary ventilation^20^ or overestimations of up to 10% using a two-regression model based on counts have been reported^44,45^.

Respiration variables seem very promising in the accurate estimation of daily TEE in comparison with other physiological or physical variables. Measuring daily TEE for different activities (e.g., cycling or strength training) based on acceleration is challenging, without a set of measurement devices with one placed on each of several body parts^35^. In contrast, respiration variables might change with every effort and seem to be unaffected by tasks involving only certain body parts or relating to movements that are performed with an extra load. It appears that respiration variables increase linearly with increased intensity not as happens with heart rate^14,46^. Interestingly, it seems that the relationship between respiration variables and TEE does not depend on the training status and the type of exercise. The latter was emphasised by the fact that Model 2, incorporating known activity categories, did not outperform Model 1, incorporating only respiration variables and age. This is in contrast to other studies, focused on acceleration and heart rate data for TEE estimation, stating that objective measurement tools are required to better assess activity type and intensity to increase the accuracy of TEE estimations^5^. Consequently, Model 1 is a promising algorithm with high feasibility as it does not require any user calibration or extended collection of user information.

The proposed models confirm and extend the previous findings that TEE can be estimated based on respiration variables. In general, a majority of previously published research showing the relationship between respiration variables and TEE was based on data obtained under limited conditions, such as during gait or other specific activities, with subjects that were male or only represented a small population^14,15,20^. Our study presents accurate models that apply across a broad range of ages, BMI levels, and training statuses, to both sexes, and during a variety of activity tasks in daily life. Hence, the population and activity task diversities in our study were higher^14,47^. Previously, it was claimed that the ventilation-based approach is not valid when ventilation is too low or too high and that it should be restricted to 15–50 l*min^−1^. However, the proposed models in the present study cover all aerobic intensities (respiration quotient < 1.0) with ventilation ranging from 5 up to 115 l*min^−1^. An additional strength of this study is that the development and validation of the models were performed separately with two distinct sample groups.

Nevertheless, future research is recommended to evaluate the proposed models when applied to a sample that is performing different activities and when assessed with an independent device. Effective, the presented theoretical models were developed under optimal conditions. Furthermore, it was not known whether the different activities that were grouped into the same category (i.e., defining walking, running, and cycling as cyclic activities) proceeded in the same way and were therefore comparable. It is possible that different classifications (i.e., low-, moderate-, and high-intensity activities) would have improved TEE estimation further. However, activity categories vary among previous studies^48–50^. Lastly, only aerobic activities were included for the model calculations due to a lack of valid formulas estimating TEE during anaerobic activities^36,37^. However, as a large amount of the population is insufficiently active or/nor barely reaches an anaerobic state during most of the days, one may connive at this limitation^51,52^.

The present study provides evidence that TEE can be accurately estimated based on respiration variables. Therefore, in a next step the incorporation of the present models into portable devices measuring respiration variables is needed for practical application in the future. For the ambulatory assessment of respiratory volume, Gastinger *et al*.^47^ presented a promising method which was based on two pairs of electromagnetic coils. Moreover, there are upcoming wearables (e.g. smart shirts or sensor system networks) that may assess respiratory volume and rate^16–19^. Such tools might be used to track changes in aerobic responses across the lifespan, allowing for the monitoring of patients during clinical interventions or rehabilitation programmes as well as in natural settings^17,19^. In the long term this may help to achieve health benefits, as TEE plays an important role in such processes as body weight regulation^1^.

## Conclusion

This study demonstrated the good validity of a model estimating daily TEE based on respiration variables and age in a broad population and during a wide range of aerobic activities. The analyses revealed equivalent results between the estimated and the measured TEE values. Consequently, the use of respiration variables to estimate daily TEE is highly recommended.
